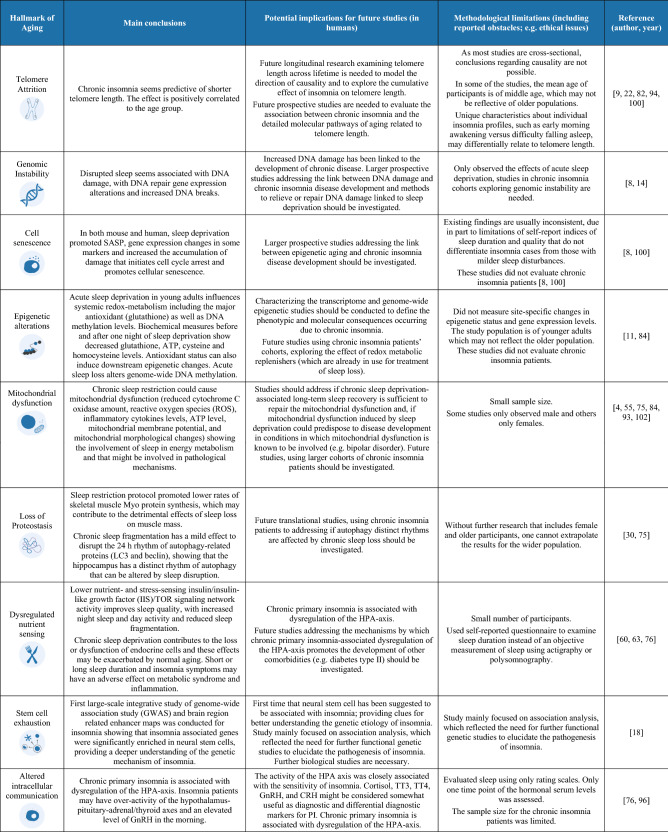# The impact of insomnia on frailty and the hallmarks of aging

**DOI:** 10.1007/s40520-022-02310-w

**Published:** 2022-12-30

**Authors:** Catarina Carvalhas-Almeida, Cláudia Cavadas, Ana Rita Álvaro

**Affiliations:** 1 CNC-Center for Neuroscience and Cell Biology, University of Coimbra, Coimbra, Portugal; 2 Centre for Innovation in Biomedicine and Biotechnology (CIBB), University of Coimbra, Coimbra, Portugal; 3 Faculty of Pharmacy, University of Coimbra, Coimbra, Portugal; 4 EIT Health Ageing PhD School and Multidisciplinary Institute of Ageing (MIA-Portugal), Coimbra, Portugal; 5 Institute for Interdisciplinary Research (IIIUC), University of Coimbra, Coimbra, Portugal

**Keywords:** Frailty, Insomnia, Hallmarks of aging, Circadian rhythms, Sleep

## Abstract

Throughout the course of life, there are age-related changes in sleep. Despite these normal changes, there is a high percentage of older adults that report sleep dissatisfaction with a high pervasiveness of chronic insomnia, the most common sleep disorder worldwide, with its prevalence being expected to continuously increase due to the growing rates of aging and obesity. This can have different adverse health outcomes, especially by promoting both physical and cognitive decline, which ultimately may aggravate frailty in older adults. Moreover, age-related frailty and sleep dysfunction may have a common mechanism related to the hallmarks of cellular aging. Cellular aging was categorized into nine hallmarks, such as DNA damage, telomere attrition and epigenetic changes. In the context of geriatric and chronic insomnia research, this review aims at discussing the current evidence from both animal models and human cohorts addressing the link between chronic insomnia, the hallmarks of aging and their impact on frailty. Moreover, the most recent research about the putative effect of insomnia therapeutic approaches on hallmarks of aging will be also highlighted.

## Introduction

As the rates of the aging population are increasing, the condition of frailty and its impact on global health burden have been gaining attention. The characteristics of physical frailty among other conditions include: increased resting energy expenditure, reduced energy intake and the prominent presence of the hallmarks of aging, which altogether contribute to an aggravated multifactorial scenario that may contribute to decrease an individual lifespan [1]. Some risk factors contributing to the onset and progression of frailty are a combination of demographic and social factors (e.g., advanced age, women, education levels, etc.), clinical factors (e.g., presence of multi-morbidity; chronic diseases, obesity, sleep disorders, etc.), lifestyle factors (e.g., physical inactivity, smoking, etc.) and biological factors (e.g., inflammation, endocrine dysregulation, etc.) [2].

Throughout the course of an individual lifespan, sleep needs also change. According to the National Sleep Foundation, normal total sleep time for people older than 65 years is approximately 6.5–7 h *per* night, which is less than the 7–9 h *per* night sleep requirements for adults [3]. Despite these normal changes in sleep total time, it is frequent for older people to report sleep problems and insomnia, in part due to sleep structure changes that naturally occur with aging that turns sleep lighter and with shorter duration [4, 5]. In the cases where sleep complaints and insomnia symptoms occur at least three times *per* week, for three months or more are indicative of chronic insomnia [6].

The physiological impact of insomnia on patients and chronic sleep loss in animal models has been long associated with premature aging and cellular-related changes that resemble aging, also described by Lopéz-Otin et al. as hallmarks of aging [5, 7]. In fact, the definition of aging can have a multitude of contexts and defining how we age is a complex question. Hence, two different concepts of aging were defined over the years in an attempt to describe and distinguish the different aging types, subdividing aging into chronological aging and biological aging. Chronological aging corresponds to the time that an individual has lived since the date of birth and, in contrast, biological aging defines the orthological and pathological characteristics of an individual at a certain time of his chronological age, which explains why individuals of the same age group have visibly different ages and may even be more susceptible than others to age-related frailty and to the early development of some diseases [8]. The concept of biological aging is marked by individual cellular alterations that occur by the combination of different hallmarks of cellular aging that altogether culminate in DNA methylation and epigenetic alterations. These alterations also known as epigenetic “clocks”, together with other internal cell changes drive to cell senescence and are considered the most accurate markers of cell aging [9, 10, 7]. In Geroscience, the measurements and different hallmarks of cell aging are also named biological cellular and molecular characteristics that help to measure biological aging, for example, an advanced biological age is representative of increased biological clock, which is translated by pronounced changes in the hallmarks of aging [7].

**Fig. 1:**
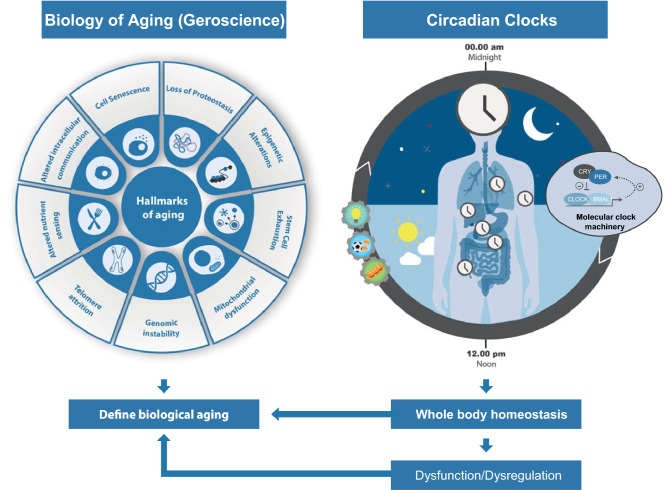
Summary of the impact of circadian clocks on biological aging in the context of Geroscience. In Geroscience, the different hallmarks of cell aging help to measure biological aging. In the context of sleep and circadian rhythms research, circadian clocks are biological 24 h period oscillations that follow a light–dark cycle and are modulated by clock genes. These clocks are also entrained by external cues such as the multiple activities the body performs during the day and night time (e.g., light exposure, feeding, exercising, sleep, etc.). Given the crucial role of circadian biological clocks in the maintenance of whole-body homeostasis, disruptions in their functioning can seriously affect functional physiology, increase disease susceptibility, and promote both biological aging and frailty

In the context of sleep and circadian rhythms research, there are circadian biological clocks, which are biological oscillations that follow a light–dark cycle of a 24 h period accompanied by a complex molecular machinery modulated by clock genes, whose functioning steadily declines throughout aging alongside the other natural changes in sleep that occur with aging [11]. Circadian clocks are located throughout our body organs with the “master clock” located in the suprachiasmatic nucleus (SCN), in the hypothalamus. These clocks regulate multiple physiological functions that are mainly performed during the day and nighttime (e.g., feeding, exercising, sleep, etc.) (Fig. 1). Given the crucial role of circadian clocks in the maintenance of whole-body homeostasis, disruptions in their functioning affect physiology, increase disease susceptibility, promote aging and decrease lifespan, as reviewed in [12]. In this context, the integrity of circadian clocks may also provide relevant and complementary insights regarding accelerated/aggravated aging.

Furthermore, the prevalence of untreated sleep problems and, in particular, chronic insomnia results in an increased burden of both direct and indirect costs incurred either by the patient or the public health system [13, 14]. The treatment of this disease seems to alleviate and improve the other medical conditions, which can have a social and economic impact [13, 14]. Thus, it is of the utmost importance to screen early the intrinsic capacity of individuals (frailty) to begin as early as possible a comprehensive geriatric assessment that allows for planning and implementation of a specific intervention to help manage an individual’s condition and promoting a patient-centered care, ensuring a dignified late life.

In this review, the important findings establishing a link between insomnia, aging, frailty, and its impact on the current nine hallmarks of aging will be discussed, particularly recently published findings within the past years will be emphasized. The current therapies (non-pharmacological and pharmacological) of insomnia will also be addressed regarding their possible interference in the hallmarks of aging.

### Circadian rhythms throughout life

**Fig. 2:**
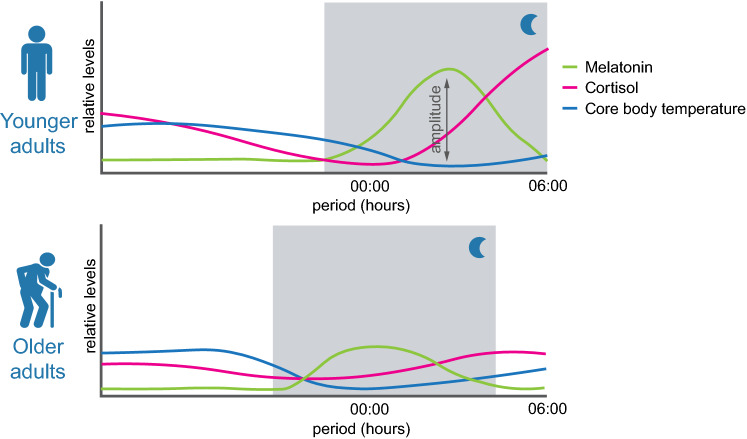
Scheme of circadian rhythms in younger and older adults of melatonin, cortisol, and core body temperature. Along the 24 h cycle several hormones, such as melatonin and cortisol, for example, follow a circadian rhythmicity, as well as body temperature. These three examples are represented, which are known to decrease the amplitude and peak expression time and relative levels, that may advance or delay with aging. Most of these changes occur naturally with aging, however, the presence of sleep disorders such as chronic insomnia may accelerate these changes. The nighttime period is represented by the shaded gray area. Throughout aging, there is a steady shift to an early chronotype, marked by a shorter sleep duration as well

Sleep is a process regulated through sleep–wake homeostasis mechanisms and by the circadian biological clocks [15, 16]. These two components of sleep regulation have been described in the past decades as the two-process model of sleep regulation, where sleep–wake homeostasis mechanisms compose the process S, and circadian rhythms are the process C [17–19]. The mechanisms of homeostatic sleep–wake regulation involve the cumulative drive for sleep during the daytime period, with adenosine being one of the key players of this process [17, 19]. The interaction between the two processes allows for the amount and timing of sleep, changes of daytime alertness and the predictive responses of body temperature, melatonin, and cortisol changes, for example, throughout the day (Fig. 2) [17, 18].

During infancy, sleep–wake rhythms are ultradian and consolidate during the first year of development. Then, a marked shift from an early to a late chronotype occurs from childhood to adolescence, subsequently shifting back to becoming early during adulthood, and with shorter sleep durations. Along with aging, circadian rhythms, such as temperature, cortisol and melatonin, also suffer a steady gradual loss with an overall delay in their peak expression, and there is a decrease in amplitude. The amplitude of rhythmic expression of some genes and hormones in the brain and other tissues is reduced during aging, affecting tissue homeostasis and function, as well as body temperature (Fig. 2), reviewed in [20]. The overall advance in the circadian rhythms phase results in both earlier bedtime and wake up time than younger individuals, reviewed in [21].

Concomitantly to these natural changes in sleep with aging, circadian biological clocks are also not resilient to the aging process, and their functioning steadily declines along this process. Many other mechanisms change together with aging, for example, neuronal communication weakens and endocrine rhythms become dampened, which compromises clocks synchronization and affects circadian gene expression, reviewed in [22, 23]. Due to the role of biological clocks in cellular and organism homeostasis, their disruptions are associated with cellular dysfunction and diseases [22]. In this context, integrity of biological clocks provides relevant and complementary insights regarding accelerated/aggravated aging. Hence, older adults may become more frail due to the disruption of sleep and circadian rhythms, as their habits also change, for e.g., they are less sensitive to light exposure, often have a sedentary lifestyle, extended naps may occur more frequently, contributing to a desensitization of the 24 h light–dark cycles [24].

### Insomnia, frailty, and premature aging

The physiological impact of insomnia and chronic sleep loss in animal models as well as cellular-related changes have been long associated with premature aging. The presence of sleep problems and insomnia disorder becomes more prevalent in older adults due to sleep structure changes that naturally occur with aging that turn sleep lighter and with shorter duration in older adults, particularly the prominent diminishing in slow wave sleep, which is of relevance for brain clearance, metabolic health homeostasis and for memory consolidation [25–27]. Neural activity may also change throughout life and particularly decline with chronic sleep deprivation. Also, it is frequent for people to report sleep dissatisfaction with aging [4, 5]. In particular, among the individuals above 65 years, it is estimated that about 12–20% suffer from insomnia disorder, which is considered the most common sleep disorder and its prevalence is expected to continuously increase due to the growing rates of aging and obesity [28, 29].

Insomnia is defined by persistent sleep difficulty, adequate sleep opportunity and associated daytime dysfunction and by sleep onset insomnia (difficulties in falling asleep), maintenance insomnia (difficulty staying asleep) and terminal insomnia (early morning awakenings), in which patients may have trouble falling asleep, staying asleep, or both [30, 31]. If one or more of the above-mentioned components are verified along with having sleep difficulty at least three times *per* week for at least three months, a patient will be diagnosed with chronic insomnia disorder [31]. Moreover, sleep duration, either short sleep duration (< 6 h *per* night) or average sleep duration (6 to 8 h), can be used to stratify insomnia patients as the number of total sleep hours correlates with insomnia severity [32]. Individuals with chronic insomnia with short sleep duration usually have genetic predisposition, physiological hyperarousal, impaired neurocognitive function, longer duration of insomnia, increased risk for cardio-metabolic diseases, hypertension, diabetes, altered heart rate variability and higher risk of mortality, thus, being considered the most biologically severe phenotype of the disorder [26, 32]. Besides poor sleep quality, insomnia patients usually report daytime fatigue and sleepiness, muscle tension, headaches, concentration problems, memory impairment and are more likely to have other comorbidities, including psychiatric and cardiovascular diseases, which strongly impacts on quality of life and may contribute to the declining of intrinsic capacity, which according to the WHO definition is the combination of both physical and mental capacities of a person at any life stage [30, 29, 31]. In fact, a bi-directional relationship between frailty and insomnia has been investigated recently, showing that poor sleep quality contributed to the onset and worsening of frailty and that this association may vary by sex and by the levels of physical activity [33, 34]. The link between insomnia and aging may promote changes in the immune system by triggering a chronic low-grade inflammatory state in aged individuals, a process also described as inflammaging [35–37]. The shift from homeostasis to low-grade inflammation promotes inflammatory responses and its severity is a predictive cause of mortality in older adults [38]. When a low-grade inflammatory state is activated chronically, it might become difficult to return to a homeostatic state during sleep repair which in turn, contributes to the development of pathologies in older persons [39, 40]. Altogether, this scenario may promote the progression of frailty and multi-morbidity.

### Insomnia and the hallmarks of aging: Rationale and evidence in the literature on animal models and humans

The process of cellular aging has been characterized by cellular alterations that have been categorized into nine biological hallmarks of aging that include: genomic instability, telomere attrition, loss of proteostasis, epigenetic alterations, deregulated nutrient-sensing, mitochondrial dysfunction, stem cell exhaustion, cellular senescence and altered intracellular communication (endocrine, neuroendocrine and neuronal) [7, 41]. Understanding how chronic insomnia contributes to each one of these hallmarks may help to find connections between sleep disorders, accelerated aging, physical and cognitive decline that could promote the development of age-related disorders and may open new avenues to decrease or help to better manage frailty. Moreover, the association between these hallmarks and sleep disorders has already been reviewed in the context of Obstructive Sleep Apnea (OSA), another highly prevalent sleep disorder worldwide, showing strong connections between these hallmarks and OSA and demonstrating evidences of premature aging in some studies in OSA patients [42]. Here, we provide a discussion on the current literature describing the impact of insomnia on each one of the current hallmarks. Also, we present a summary of the current findings reported for each hallmark in Box 1.

#### Telomere attrition

Telomeres are repetitive nucleotide sequences located at the ends of chromosomes that protect them from deterioration or fusion with neighboring chromosomes, being particularly fragile to age-related changes. Throughout the course of life, telomeres length tends to shorten and, despite this normal shortage, many studies have associated both telomere dysfunction and telomerase deficiency (the enzyme that maintains this region) with age-related diseases and premature aging in mice and humans, as an estimate of accumulated cell damage and aging [43, 42, 44]. The link between telomere length and insomnia or sleep deprivation has been reported, with cross-sectional and longitudinal studies showing associations between insomnia symptoms, objective sleep duration and chronic insomnia with shorter telomere lengths [45, 43, 46, 47, 44]. In 2014, the first link between telomere length and insomnia was observed in a cohort of breast cancer post-menopausal survivors with severe insomnia, where shorter telomere length was positively associated to insomnia compared to the control group (age and BMI-matched women without insomnia), however, this difference was not statistically significant [43]. Subsequently, other authors investigated this association and have observed similar results in different insomnia cohorts with older subjects and middle-aged adults [45, 46]. A cross-sectional study evaluating older adults with insomnia reported an association between insomnia and short telomere length in individuals aged 70–88 years, but not in the 60–69 years study group. These observations may indicate that clinically severe insomnia may anticipate and/or increase vulnerability to cellular aging, particularly in the later years of life, with implications for risk of developing age-related diseases [45].

However, the correlation between telomere length and disease severity (stratified according to objective sleep duration) remains controversial and further studies are necessary [46, 44]. For example, it was found an association among insomnia, insomnia phenotype, and self-reported long sleep duration with the maintenance of telomere length [46]. By contrast, in a longitudinal study, the authors uniformly show that delayed circadian rhythm is associated with shorter telomere length and not short sleep duration or insomnia [47]. The difference reported by both studies may be in part explained due to differences in study cohort population, namely on the age and BMI of the subjects, the technique used to measure telomere length (although they both used leucocytes as a sample) and the methods/guidelines used to diagnose insomnia disorder.

On a side note, the association between telomere dysfunction and chronic sleep deprivation has also been observed in mice [44]. In summary, it seems that telomere length might be affected by sleep loss, however, future longer longitudinal basic and clinical studies are needed to further understand both the relationship between insomnia, objective sleep duration and telomere length and the biological mechanisms underlying this association that could be promoting early aging.

#### Genomic instability

Genomic instability refers to the accumulation and exposure of exogenous and endogenous threats (e.g., oxidative stress), which impact on DNA integrity and stability [42]. DNA damage can be induced by sleep deprivation. In 2015, a study involving individuals over 60 years old showed that one night of sleep deprivation was sufficient to induce DNA damage response [48]. Later, in 2019, Cheung and colleagues showed that acute sleep deprivation in a middle-aged shift workers community increased DNA break damage and reduced the expression of DNA repair genes [49]. Both studies highlight that disrupted sleep is associated with DNA damage and that acute events of sleep deprivation are sufficient to induce this damage. Nonetheless, studies evaluating the impact of chronic sleep deprivation on genomic stability are needed. Also, the impact of DNA damage and chronic disease development still requires further investigation, namely, the proposed pathways triggering such outcomes and whether the administration of antioxidants or the recovery of sleep after sleep deprivation can repair this damage.

#### Cell senescence

Cellular senescence is a state where the cells stop dividing, usually due to replicative issues, such as telomere shortening or activation of cell stress and DNA damage pathways, causing a signaling cascade of DNA damage response elements, which drives to cell cycle arrest [48, 42]. The accumulation of senescent cells is thought to promote/aggravate the process of biological aging and be predictive of a shortened lifespan. These senescent cells are characterized by a specific secretory pattern, also known as the senescence-associated secretory phenotype (SASP), which promotes the increased release of inflammatory cytokines and chemokines [48, 42]. There is evidence that acute sleep deprivation is sufficient to increase the expression indicative of SASP, and senescence indicator p16^INK4a^ (*CDKN2A*) in older adults [48]. In accordance, a cross-sectional study evaluating post-menopausal older woman shows that insomnia symptoms and short objective sleep duration are predictive of accelerated aging, as showed by increased immune cell senescent markers [50]. These findings are also observed in sleep-deprived mice that exhibit increased levels of senescence-associated cytokines secretion and subsequent SASP [44]. However, the detailed mechanisms by which this occurs remain to be investigated and future studies investigating the correlation between cell senescence and chronic insomnia, instead of acute events of sleep deprivation are needed to reveal senescence-associated pathways and the putative causative and/or mechanistic roles of chronic insomnia.

#### Epigenetic alterations

Epigenetic alterations have been referred to as an alternative biomarker of aging, based on DNA methylation (DNAm) also known as the epigenetic clock, which provides an estimate for chronological age across cell types and complex tissues by capturing aspects of biological age. Besides DNA methylation, other epigenetic alterations include post-translational modifications of histone proteins, chromatin remodeling, and transcriptional regulation by noncoding RNAs, which can impact on cell and tissue homeostasis. It is thought that approximately 40% of epigenetic age acceleration is due to inheritable causes (endogenous factors), while the remaining 60% is due to exogenous factors [42, 50]. Short-term sleep deprivation has been linked to causing DNA hypo-methylation in plasma from young healthy adults, possibly due to the activation of oxidative stress signaling pathways and ATP decrease [51]. A study in a cross-sectional cohort of older woman post-menopause also observed a correlation between insomnia symptoms and epigenetic age, measured by DNA methylation levels. They also predicted that both short and long sleep duration are correlated with older epigenetic age [50].

Future studies addressing this hallmark in the context of chronic insomnia in a larger population and studying also older adults may elucidate the impact of this hallmark on aging and the underlying risk for developing other comorbidities.

#### Mitochondrial dysfunction

Throughout the course of cell aging, mitochondria homeostasis can change by either altering their normal function or biogenesis, which decreases the efficacy of the respiratory chain, reduces ATP levels, promotes leakage of electrons and reactive oxygen species (ROS), as result of enhanced oxidative stress. Alterations in mitochondrial activity and increased oxidative stress have been linked to trigger early-aging mechanisms and to underlie many diseases. Sleep loss has been proposed as an enhancer of oxidative stress and promoter of mitochondrial dysfunction. In accordance, sleep-deprived mice have been shown to have decreased mitochondrial complexes activity and that mitochondrial dysfunction is involved in the regulation of sleep recovery [52, 53]. Also, in humans, sleep loss has been shown to impact mitochondrial function and one night of acute deprivation is sufficient to induce redox-based global DNA methylation changes in plasma samples, decrease the levels of plasma antioxidant GSH, elevate oxidative damage and decrease ATP levels. These changes may be underlying the pathogenesis of neurological diseases [54, 51]. Besides the acute effects of sleep deprivation, chronic sleep restriction also results on mitochondrial dysfunction shown by morphological changes in these organelles in the in the frontal cortex of mice, decreased mitochondrial respiratory chains complex concentration, low ATP levels and mitochondrial membrane potential. Furthermore, chronic sleep deprivation seems to promote a mitochondrial-dependent amyloid *β* (Aβ) accumulation in the cortical region, which is one of the hallmarks of the physiological changes that occur in Alzheimer’s disease [55]. Nonetheless, little is known about the link between insomnia and mitochondrial dysfunction. Recently, new evidence trying to connect insomnia with mitochondrial dysfunction in a small group of post-menopausal female patients (age 55–70 years) suffering by either objective or paradoxical insomnia, shows that both forms of insomnia elicit a stress response involving myokines’ production (FGF21, GDF15 and HN, molecules that expressed in response to mitochondrial stresses) [56]. Thus, insomnia may contribute to mitochondrial dysfunction, a hypothesis that should be investigated in future, possibly in larger cohorts involving both genders and different age groups. The underlying mechanisms responsible for mitochondrial dysfunction induced by insomnia remain unknown and it is unclear how such impairments could contribute to aging or age-related disorders. However, it seems that insomnia might be activating mitochondrial stress-related pathways and exacerbating oxidative stress production.

#### Loss of proteostasis

Proteostasis refers to the process of protein homeostasis that involves many steps from protein synthesis, folding, aggregation, to degradation [42]. Within the cell, the endoplasmic reticulum (ER) is the site of secretory and membrane protein synthesis that contains the necessary machinery necessary for proteostasis. In the ER lumen, the newly synthesized proteins bind to the molecular chaperones that allow them to fold in their appropriate conformation. After, they need to exit the ER to perform their function within the cell. When proteins are not properly folded, they are tagged to the proteasome for degradation. Despite the presence of molecular chaperones and multiple checkpoints, some misfolded proteins will accumulate in the lumen of the ER. Several stressors such as sleep perturbations may trigger proteostasis impairment due to increased oxidative stress, hence, contributing to aging and age-related diseases by causing an excessive accumulation of proteins [42, 7]. Recent evidence shows that the proteolytic systems are regulated in a circadian rhythm pattern with an interplay between the circadian clock and chaperone-mediated autophagy (CMA), which allows for the circadian remodeling of the cellular proteome. Hence, perturbations in the autophagic pathway in vivo causes temporal and amplitude shifts of clock-dependent transcriptional waves and causes the fragmentation of circadian patterns, resembling those in sleep disorders and aging [27]. Also, the circadian rhythm of autophagy proteins is blunted by chronic sleep fragmentation in the mice hippocampus, as determined by an arrhythmic expression of LC3-II protein [57].

Besides the molecular pathways of protein synthesis, sleep has also been implicated in the maintenance of muscle mass with individuals reporting short objective sleep duration or poor sleep quality having an increased likelihood of both sarcopenia and lower total skeletal muscle mass, which can impact on physical frailty [58, 54]. Overall, muscle mass is influenced by the balance between the rates of muscle protein synthesis and muscle protein breakdown. Recently, it was investigated in humans the potential mechanisms by which sleep restriction may contribute to reduction in muscle mass. Individuals that followed a 4 h *per *night sleep restriction protocol, for five nights (with or without three sessions of high intensity interval exercise) showed significantly lower rates of Myo protein synthesis (MyoPS) compared to the non-sleep restricted groups. Also, the authors reported a dissociation between the changes in MyoPS and the molecular signaling pathways that regulate proteostasis. Due to the growing prevalence of sleep problems in society and the importance of the skeletal muscle for physical robustness, it becomes urgent to understand the mechanisms and implications of chronic insomnia in muscle mass [54]. Despite this important evidence, to our knowledge, no studies have yet explored the correlation between the effects of chronic insomnia or chronic sleep deprivation in animal models and the role of proteostasis.

#### Dysregulated nutrient-sensing

Nutrient sensing pathways have been reported to become deregulated and lose effectiveness with age. The insulin and insulin-like growth factor1 (IGF-1) signaling pathway together with its downstream targets, mTOR, AMPK, and Sirtuins constitute the most evolutionarily conserved nutrient-controlling pathways, affecting multiple targets and have been associated with longevity in both humans and animal models [42, 7]. These pathways constitute a direct link between diet and aging with dietary caloric restriction being associated with increase in lifespan and health span in all investigated models, including non-human primates [7]. Similarly, over the past decade, several studies have suggested a bi-directional link between sleep deprivation and metabolic dysfunction, evidencing possible deregulated nutrient-sensing pathways, such as alterations in glucose metabolism and insulin resistance. Both sleep duration and insomnia symptoms have been linked with components of metabolic syndrome and inflammation in middle age and older patients with metabolic syndrome [59–61]. Moreover, acute sleep loss seems to be involved in tissue-specific changes and the differential use of anabolic or catabolic pathways [58]. Altogether, aging and sleep deprivation seem to promote unfolded protein response in the different organs such as the pancreas with implications for metabolism [62]. In chronic sleep-deprived young mice, it was reported sensitization to insulin and better glycemic control, while aged animals were hyperglycemic and had reduced insulin concentration levels in the plasma [62]. In drosophila, the diminishing of insulin signaling via insulin/insulin-like growth factor /TOR signaling pathway has been proven to ameliorate age-related sleep fragmentation. A similar decline in sleep quality is also observed during aging in this organism. This may occur possibly via different day and night mechanisms [63]. In humans, unfortunately, only a few studies have explored the mechanisms and effects of nutrient-sensing in chronic insomnia patients. In 2013, a study performed in older post-menopausal woman diagnosed primary chronic insomnia demonstrate a dysregulation of circadian cortisol night secretion, however, they were not associated with impaired metabolism of glucose and lipids. Nonetheless, given the damaging effects of cortisol over secretion in metabolism, the nocturnal rise in cortisol levels may increase the risk to develop diabetes and dyslipidemia [64]. Furthermore, a systematic review addressing the metabolic rate in adult patients with insomnia reported slight increase in oxygen consumption across 24 h in insomnia patients compared with healthy sleepers. Nonetheless, the clinical significance of these differences is still unknown [65]. Larger, methodologically robust studies are required to confirm these findings and the effect of any increase in metabolic rate on the pathophysiology of chronic insomnia and by which mechanisms this occurs.

#### Stem cell exhaustion

Stem cell exhaustion refers to the decrease in the capacity of stem cell pools to proliferate, differentiate and/or self-renewal into organ and/or tissue-specialized cells. As we age, these alterations can be triggered by exacerbated oxidative stress and inflammation, which can cause detrimental consequences. For example, alterations in neural stem cells (NSCs) can be connected to age-related cognitive decline [42, 7]. Evidence evaluating this hallmark in chronic insomnia or chronic sleep deprivation models is scarce. So far, literature on acute sleep deprivation suggests that Rapid Eye Movement (REM) sleep deprivation promotes loss of neurogenesis in the hippocampal dentate gyrus (DG) in adult rats, suggesting a role for REM sleep-associated processes in contributing for the proliferation of granule cells in the adult hippocampal DG [66]. In fact, a role for sleep and adult DG neurogenesis has been reviewed by Koyanagi 2019 [67]. More recently, a study evaluating acute REM sleep deprivation in mice, found a possible mechanism contributing to the inhibition of cell proliferation in the DG by inducing the IL-17/p38 MAPK pathway [68]. Moreover, a protocol for long-term sleep deprivation in mice described a role for melatonin in the recovery of SOX^2+^, a marker of cell proliferation, in the DG. Also, mice injected with melatonin under long-term sleep deprivation had higher levels of MECP2 expression and reduced the SIRT1 expression in the DG, two molecules involved in DNA methylation and cell longevity and differentiation, respectively. These findings may contribute to further understanding of the possible mechanisms of chronic sleep deprivation on neural progenitor cells proliferation [69]. Furthermore, a research study conducted an integrative analysis of genome-wide association study (GWAS) and brain region-related enhancer maps showed that insomnia associated genes (MADD, CASP9, PPP2R3C and, PLEKHM2) were significantly enriched in neural stem cells, highlighting a set of potential points which suggests that neural stem cells may contribute to insomnia via regulating the generation of sleep-waking switch-related neurons. In future, neural stem cells should be considered in future biological studies for insomnia [70]. Also, it should be explored in future studies how chronic insomnia may impact other stem cell niches, such as endothelial progenitor cells (EPCs) or mesenchymal stem cells (MSCs), which have been addressed in other sleep disorders, including obstructive sleep apnea [42].

#### Altered inter- and intracellular communication

Alongside the aging process, there are also inter- and intracellular communication changes either at the endocrine, neuroendocrine, or neuronal level. These changes tend to be accompanied by neuroendocrine signaling dysfunction (e.g., renin-angiotensin, adrenergic, insulin-IGF-1 signaling), exacerbated inflammatory responses and immune surveillance vulnerability, as well as changes in peri- and extracellular composition and environment that may challenge intercellular communication [42, 7].

Studies in patients with chronic insomnia and objective short sleep exhibit altered insulin-IGF-1 signaling, reporting lower levels of plasma insulin, which could increase the predisposition to develop type II diabetes [71]. Also, patients with primary chronic insomnia reported higher midnight salivary cortisol concentrations, which is associated with dysregulation of the hypothalamic–pituitary–adrenal (HPA) axis and has been demonstrated to impact glucose metabolism although no changes in glucose and lipid metabolism were detected in this study group, nocturnal cortisol over secretion may increase the predisposition to develop diabetes and dyslipidemia [64]. Other studies have also found increased levels of pituitary, adrenal, thyroid and gonadal axis hormones (adrenocorticotropic hormone (ACTH), cortisol, and thyroid stimulating hormone (TSH), corticotropin-releasing hormone (CRH), thyrotropin-releasing hormone (TRH), Gonadotropin-releasing hormone (GnRH), total thyroxine 3 and 4 (TT4, and TT3)) and catecholamines (norepinephrine and epinephrine) in chronic insomnia patients, which seem to be correlated with insomnia severity [72, 64, 73]. All in all, insomnia disorder seems to promote over-activation of the HPA axis, which causes dysregulation and may constitute a pathway underlying adverse health consequences in the long-term and contribute to early aging [72, 74, 73].

Furthermore, the accumulation of alterations in signaling pathways tends to exacerbate inflammatory reactions, thereby promoting the accumulation of inflammatory changes and a dysfunctional immune system (inflammaging) [7]. Sleep loss in humans seems to culminate in the over-activation of the NF-kB pathway which is one of the transcriptional signatures of aging that serves a critical role in the inflammatory signaling cascade [25, 7]. Although to our knowledge no studies have directly investigated the activation of this transcription factor in chronic insomnia, the relevant pro-inflammatory cytokines (e.g., C-reactive protein, IL-1β, IL-6, IL-8, and TNF-α) and chemokines associated with this pathway have been reported to be increased in the blood plasma, serum and saliva of chronic insomnia patients [75, 76]. Curiously, the same findings were described in a rat model of aging insomnia [77]. Given the necessity of new models to investigate insomnia pathophysiology, it would be interesting to evaluate the impact of the hallmarks of aging in such models and investigate potential therapeutic strategies that could target these hallmarks and evaluate the progression of aging.

On a last note, beyond the accumulation of inflammation, dampened intra- and intercellular signaling altogether can also impair organ-to-organ communication and have implications on age-related functional defects [7].

### Do the current insomnia therapies impact the hallmarks of aging?

Individuals suffering from chronic insomnia are more likely to suffer from other comorbidities and psychological consequences, specially, when left untreated, which reinforces the importance of treating this disorder. According to the 2017 European guidelines for insomnia treatment, the most common clinical intervention for chronic insomnia treatment relies on cognitive behavioral therapy for insomnia (CBT-I) and other psychotherapeutic approaches. Besides, other complementary non-pharmacologic approaches may be used as adjuvant therapies [78].

#### Cognitive behavioral therapy for insomnia (CBT-I) and other psychotherapeutic approaches

CBT-I uses a multiple combination of approaches that involve the implementation of sleep hygiene rules (e.g., health practices that improve sleep duration and quality, such as avoiding exposure to blue lights from electronic devices before bed or having a sleep/wake schedule routine), psychoeducation (e.g., education about normal sleep and age-related changes in sleep architecture), relaxation techniques (e.g., breathing exercises, mindfulness), stimulus control therapy, sleep restriction therapy and cognitive therapy [78].

Evidence from studies evaluating the effect of CBT-I and other psychotherapeutic approaches impact on the hallmarks of aging in patients with chronic insomnia cohorts is scarce. Nonetheless, studies exploring the effect of these treatments on other diseases, such as depression, anxiety, stress and adjustment disorders, show that practicing mindfulness-based group therapy for eight-weeks as a CBT-I strategy is unable to alter leukocyte telomere length [79]. In addition, the use of venipuncture as a CBT/psychotherapeutic treatment for social anxiety disorder for 9 weeks did also not translate in changes in telomere length and telomerase activity post treatment [80]. Moreover, electroconvulsive therapy treatment for severe treatment-resistant depression did not alter telomere length in depression patients as well [81]. Altogether, the evidence shows that the telomere attrition marker seems unaltered with CBT and psychotherapeutic approaches in many psychological disorders and future studies using patients with chronic insomnia should be addressed.

In contrast, the practice of relaxation techniques in sleep disorders seems to be linked to changes in some age-related parameters although the underlying molecular mechanisms remain vastly unknown. However, evidence from healthy practitioners either with vast experience or beginners seem to enhance the expression of genes associated with mitochondrial function, energy metabolism, insulin secretion and telomere maintenance and reduce the expression of genes related to pro-inflammatory and stress responses, including NF-kB upstream and downstream targets [82]. Further studies, exploring telomere length and the other aging hallmarks in chronic insomnia patients in older adults following these treatments should be investigated.

#### Pharmacological approaches

When CBT-I and other psychotherapeutic strategies cannot be applied or are not sufficient to promote positive alterations in chronic insomnia patients, there are also pharmacological options. In Europe, the mostly used drug classes used to treat insomnia disorder include benzodiazepines, benzodiazepine receptor agonists, antidepressants, antipsychotics, antihistamines, psychotherapeutic substances and melatonin [83].

The use of benzodiazepines and benzodiazepine receptor agonists has a rapid and potent effect, but its use is also associated with negative outcomes as well, such as causing physical dependence, promoting withdrawal syndrome and cognitive impairment. However, not much is known about the effects of their prolonged use on the hallmarks of aging or by which mechanisms they can impact on cellular aging. Nonetheless, the use of Diazepam, which is a benzodiazepine compound used for the treatment of insomnia has been suggested to promote oxidative stress damage and to be genotoxic by inducing chromosome changes in a dose-dependent manner in a human in vitro lymphocyte culture [84]. In rodent animal models, the prolonged treatment of Diazepam caused lipid peroxidation and protein carbonyl formation in the brain and diminished the levels of glutathione and superoxide dismutase activity in the liver [85, 86].

Furthermore, the use of benzodiazepines in geriatrics and, in particular, their prolonged use is not recommended because of increased likelihood of falls and, as previously mentioned, to cause further disruption of sleep, dependence problems and withdrawal [87, 88]. In a recent review, addressing the risks of benzodiazepine treatment for insomnia in older patients, the authors conclude that the older adults should ideally be treated with a non-pharmacological approach as first line treatment and, if a pharmacological approach is required, one should consider sleep aids that are not benzodiazepines or benzodiazepine receptor agonists [88].

Complementary non-pharmacological interventions.

Other complementary strategies may include light therapy and exercise that have been shown fruitful in the treatment of patients with insomnia. Besides sleep, the practice of physical exercise impacts on psychological and physical health and its regular practice may even reduce mortality. Also, alternative medicines have been tested, such as acupuncture, acupressure, aromatherapy, foot reflexology, homeopathy, meditative movement therapies, moxibustion, music therapy and yoga [83]. However, they are not part of the current guidelines for insomnia treatment and are only recommended as adjuvant therapies, partly because most evidence comes from basic and public health research and there is a lack of studies proving its relevance in the treatment of patients with chronic insomnia [83].

Physical exercise is one of the most addressed lifestyle factors that seem to have a multifactorial effect, impacting quality of life, mental health and allowing to reduce and delay age-related comorbidities and frailty. A review evaluating the effect of physical exercise on the nine hallmarks of aging shows an amelioration on all the hallmarks, giving also general guidelines for physical exercise in older people [89]. Moreover, a study performed in sleep restricted humans that followed a high intensity interval exercise protocol showed an improvement on glucose tolerance, mitochondrial function, sarcoplasmic protein synthesis, as well as an improvement on diurnal rhythms [54]. In line with these findings, others have reported the effects of exercise on serum metabolites of middle-aged individuals with chronic insomnia and sleep apnea that completed a 6-month intervention of exercise sessions, showing altered expression of 21 metabolites. From these metabolites, 7 were significantly correlated with sleep parameters (alpha-ketoisocaproic acid, 3-hydroxybutyric acid, D-glucopyranose, Tagatose, Oxalic acid, 4-deoxyerythronic acid, 2-keto-isovaleric acid) that are involved with carbohydrates, lipids, and organic acids metabolisms. Nonetheless, more evidence is needed to determine causality between these changes and the underlying molecular pathways that are due to lifestyle changes, such as physical exercise or even dietary interventions [90].

The impact of diet may influence insomnia and insomnia symptoms. For example, caffeine consumption and other dietary behaviors may be contributing to the aggravation of insomnia [91]. Although the literature is not consistent, it seems that the macronutrient distribution of our diet can contribute to the aggravation or alleviation of insomnia symptoms and, an unbalanced diet may predispose for more severe insomnia [92]. The effect of different nutrients on sleep and sleep disorders has been reviewed elsewhere [91]. However, the effect of a healthy diet intervention or the effect of weight loss as a complementary non-pharmacological approach to treat chronic insomnia remains to be elucidated. Nonetheless, some evidence from a six-month dietary caloric restriction intervention among overweight and obese men with chronic insomnia showed that reducing the daily caloric intake by 300–500 kcal, helps improving shorten sleep onset latency and total sleep time [93]. Additionally, a study addressing 471 Japanese men showed an association between insomnia symptoms and dietary intake deficiency in fiber, vitamin C and zinc [94]. Others, evaluating the United States population, relate sleep perturbations with nutrient deficiencies in carbohydrates, vitamin C and calcium [95]. In fact, vitamin C seems to be an important and promising nutrient linking sleep with diet and, the deficiency of this vitamin might cause short sleep duration and poor sleep quality, possibly due to increased oxidative stress [95, 94, 96]. Studies using chronic sleep-deprived rodents show that supplementation with vitamin C helps prevent spatial memory impairment and improves the levels of different antioxidant biomarkers [96]. Hence, although we cannot pinpoint any specific nutrient, the supplementation of nutrients (e.g., vitamins) may be a fruitful strategy for insomnia symptoms management and should be addressed in future research. In sum, the effects of a balanced healthy diet with appropriate macronutrient, vitamins and mineral intake, caloric restriction and weight loss could also be relevant complementary approaches to alleviate insomnia symptoms, which are of low cost and easy adherence [97].

Collectively, these complementary therapies, such as light therapy, physical exercise (and other physiological stressors), and the time of food (and caffeine) intake, are contributing to the modulation of circadian function [98]. Hence, the observed beneficial effects of these therapies may be due to the possible resynchronization of circadian rhythms or through the improvement of circadian parameters. In support of this, morning bright light therapy in institutionalized older adults seemed to improve parameters of circadian rhythms, sleep, health, and cognitive factors [99, 100]. The effects of forced physical exercise in mice was shown to resynchronize the rhythms of peripheral clocks [101, 102]. Besides, one should also consider the timing of the exercise impact on circadian alignment [103, 104]. Additionally, the alignment of mealtime, calorie restriction and fasting has also been shown to promote resynchronization of circadian rhythms, as well as being associated with extended life span [105–107].

Together with nutrition and physical exercise, cognitive training and social participation are all part of the four pillars of healthy aging according to the World Health Organization 2002 and updated by the International Longevity Centre in 2015 [108, 109]. The effects of sleep deprivation are associated with memory impairment and cognitive dysfunction. Individuals with chronic insomnia usually have premature cognitive function decline and have a higher predisposition for the development of Alzheimer’s disease [110]. Emerging evidence shows that cognitive training in older adults helps to manage insomnia symptoms, which helps to tie a link between the importance of a healthy sleep for a good cognitive performance. In fact, the standard treatment for insomnia (CBT) has been tested in a randomized pilot clinical trial to enhance cognitive function and preliminary findings suggest that CBT may reduce the rate of Aβ deposition in older adults with insomnia and potentially delay Alzheimer’s disease onset [110]. However, whether other types of cognitive training should be used as complementary insomnia non-pharmacological treatments remains to be elucidated [111, 112].

Regarding social participation, it refers to the enrollment and participation in activities that require interaction with others. As social participation usually decreases with aging, due to retirement, death or illness among friends and family, reallocation and other impacting life events, health conditions and socio-economic status can also have an impact on sleep and sleep quality. Recent reports on older communities showed that people experiencing social isolation, loneliness and having less social support were more likely to suffer from insufficient sleep, poor sleep quality and social disturbances [113–115]. Due to the impact of both loneliness and insufficient sleep for mortality and morbidity in older people, it is essential that opportunities for social participation in the older population are provided, for example, by implementing comprehensive public health approaches, with public health policies that maximize positive trajectories of aging and that help reducing the limits for the participation and contribution of older adults and address their needs [116]. Also, evidence suggests that improving sleep may be a promising approach to ameliorate mental health and, thus, improve social activity among older adults [115]. Hence, raising public awareness about the importance of healthy sleep and about sleep hygiene rules becomes pivotal.

## Concluding remarks

Overall, several studies have contributed to our understanding of the pathophysiology mechanisms of chronic insomnia and its outcomes on the hallmarks of aging, which impacts the quality of life, lifespan and the development and progression of many age-related pathologies. Here, we provide the first review collecting evidence on the hallmarks of aging that seem to be involved in chronic insomnia. In fact, several of the hallmarks suggest that chronic insomnia contributes to the aggravation of cellular aging, namely, telomere attrition, deregulated nutrient-sensing, mitochondrial dysfunction, cellular senescence and altered intercellular communication, as summarized in box 1. Nonetheless, only a few studies connect each hallmark to chronic insomnia or chronic sleep loss animal models and so, future studies are needed to validate this connection. Besides the few existing studies connecting each hallmark and chronic insomnia, it is important to note that the included studies in this review were highly heterogeneous in terms of design (case–control; cross-sectional cohort studies), demographic characteristics (some with only men included, others only women and others both; only older adults versus all age ranges) or health status (e.g., breast cancer survivors). Also, the included studies addressing the acute effects of sleep deprivation may shed light onto the mechanisms that could be dysfunctional in more severe and prolonged cases of sleep deprivation, as well as its cumulative damage that may be contributing to accelerated aging.

Moreover, future research addressing targets of the hallmarks of aging as potential therapeutics to ameliorate insomnia symptoms in chronic insomnia patients should be investigated. So far, for example, strategies to restore defective intercellular communication that include many possibilities, such as genetic, nutritional or pharmacological interventions, may improve cell–cell communication properties that are lost with aging [7].

**Fig. 3:**
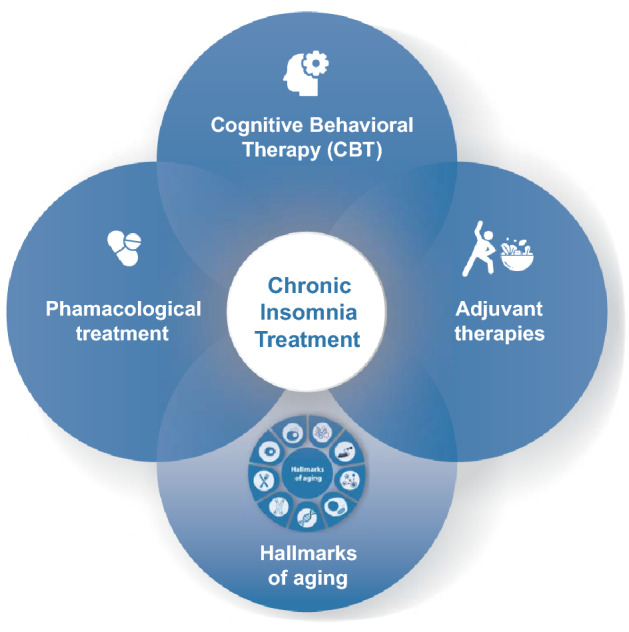
The current strategies for chronic insomnia treatment and the link with hallmarks of aging. The gold-standard treatment for chronic insomnia is cognitive behavioral treatment, which involves for e.g., implementation of sleep hygiene rules, psychoeducation, relaxation techniques, stimulus control therapy, sleep restriction therapy and cognitive therapy. There are also a range of pharmacological options which include benzodiazepines, antidepressants, antipsychotics, antihistamines, psychotherapeutic substances, and melatonin. Other adjuvant approaches include light therapy, exercise, caloric restriction, healthy diet, cognitive training or social participation. Altogether these strategies may prove fruitful in alleviating chronic insomnia symptoms and may have a synergistic effect on aging progression namely, by impacting the hallmarks of aging

Regarding the current treatment options and their impact on the hallmarks of aging, less evidence correlates these two. Possibly, the combination of the current therapies and the better management of insomnia symptoms might show a synergistic effect that may impact these hallmarks but, more studies in this field are needed (Fig. 3). In future, other complementary non-pharmacological approaches for insomnia management and their impact on aging should be deeply explored, including the effects of physical exercise, nutrition, cognitive training and social participation which are the four pillars of healthy aging [108, 109].

On a last note, as sleep problems and circadian rhythms alterations become more and more prevalent in society, in future, chrono-disruption, meaning circadian rhythm dysfunction together with sleep disruption, could be pinpointed as an additional hallmark of aging, as suggested in a review that addresses a paradigm shift on the current hallmarks [117].

Box 1 Summary of the studies addressing the hallmarks of aging in both human and animal studies of either acute or chronic sleep deprivation/insomnia.